# Proliferation and apoptosis studies of interplacental areas after aglepristone treatment for planned cesarean section in pregnant bitches

**DOI:** 10.14202/vetworld.2024.956-962

**Published:** 2024-05-04

**Authors:** Chunsumon Limmanont, Suppawiwat Ponglowhapan, Paisan Tienthai, Preeda Lertwatcharasarakul, Thareerat Sathaphonkunlathat, Kaitkanoke Sirinarumitr

**Affiliations:** 1 Department of Companion Animal Clinical Sciences, Faculty of Veterinary Medicine, Kasetsart University, Bangkok, 10900, Thailand; 2 Theriogenology Center, Kasetsart University Veterinary Teaching Hospital, Faculty of Veterinary Medicine, Kasetsart University, Bangkok, 10900, Thailand; 3 Kasetsart University Research and Development Institute, Kasetsart University, Bangkok, 10900, Thailand; 4 Department of Obstetrics, Gynaecology and Reproduction, Faculty of Veterinary Science, Chulalongkorn University, Bangkok, 10330, Thailand; 5 Department of Anatomy, Faculty of Veterinary Science, Chulalongkorn University, Bangkok, 10330, Thailand; 6 Department of Pathology, Faculty of Veterinary Medicine, Kasetsart University, Nakhon Pathom, 73140, Thailand

**Keywords:** aglepristone, apoptosis, bitch, cesarean section, proliferation

## Abstract

**Background and Aim:**

Progesterone (P4) is the main hormone for pregnancy maintenance, occurring approximately 62–64 days after ovulation in bitches. Progesterone acts by binding to specific receptors. Aglepristone is a progesterone receptor (PR) antagonist with a higher affinity for PR binding. There are no published studies on cell proliferation and apoptosis in the canine uterus at the time of parturition. Therefore, this study aimed to determine the local effects of aglepristone on cell proliferation and apoptosis of interplacental uterine tissue during planned cesarean section (C-section) in bitches.

**Materials and Methods:**

In this study, 13 client-owned French bulldogs were examined. Bitches were divided into treatment (n = 8) and control (n = 5) groups. Ovulation timing was predicted based on the serum P4 level on 62–64 days post-ovulation for parturition. Serum P4 levels were measured before (on 60-day post-ovulation) and on C-section day (on 61-day post-ovulation). Aglepristone (Alizine^®^), 15 mg/kg subcutaneously (SC), was administered on 60 days post-ovulation in the treatment group. A C-section was planned 20–24 h later, and interplacental uterine areas were collected from both groups during the C-section. Immunohistochemistry based on Ki-67 and TUNEL assay was used to evaluate cell proliferation and apoptosis in four different interplacental uterine tissue layers (epithelium, stroma, glandular epithelium, and myometrium). Data are reported as mean ± standard deviation. Kruskal–Wallis test was used for comparisons of more than two independent groups. P value of 0.05 was considered statistically significant.

**Results:**

One bitch in the treatment group was excluded due to emergency C-section 8 h after aglepristone administration. Serum P4 levels (ng/mL) at 20–24 h before and at C-section were 6.09 ± 2.72 and 4.32 ± 2.2 in the treatment group (n = 7) and 5.45 ± 1.28 and 3.67 ± 1.89 in the control group (n = 5), respectively. Proliferation (PI) and apoptotic (AI) indices were <5% and >45%, respectively, in both the treatment (n = 5) and control (n = 3) groups. PI and AI were detected at interplacental areas.

**Conclusion:**

There were no significant differences in serum P4 levels or PI and AI indices between the groups. The PI <5% and AI was higher than 45% in both groups. Aglepristone did not have a direct effect on the serum P4 levels in both groups. These results correlated with the natural physiology of parturition preparation. Aglepristone 15 mg/kg SC injected 20–24 h before parturition had no effect on the P4 level, nor were any harmful effects observed for a planned C-section in pregnant bitches.

## Introduction

Progesterone (P4) is the main hormone in the luteal phase (diestrus) produced by the corpus luteal cells in the ovaries that have developed post-ovulation [1, 2]. High levels of P4 are detected in both non-pregnant and pregnant luteal bitches [1–4]. P4 promotes uterine growth for implantation, placental development, and suppression of myometrial contractility in pregnant bitch [3, 5]. At the end of the diestrus stage, P4 was dropped due to luteal regression in non-pregnant bitches and luteolysis in pregnant bitches, resulting in parturition. Parturition was initiated when P4 levels decreased below 2 ng/mL [1, 6]. The effects of P4 are mediated by binding to its nuclear receptor, progesterone receptor (PR), which acts to target tissues such as the ovary, uterus, mammary gland, bone, and brain [7, 8]. PR is expressed in maternally derived decidual cells and plays a crucial role in fetomaternal communication during pregnancy maintenance. Understanding PR-mediated signaling has clinical implications for improving reproductive performance in bitches. Altering PR signaling induces prostaglandin F2 alpha release from the fetal trophoblasts and hinders placental homeostasis [9]. This phenomenon can also be achieved using antigestagens such as aglepristone [9]. One of the most important effects of steroid hormones is the proliferative capacity of the reproductive organs. Van Cruchten *et al*. [5] studied the proliferation of the canine endometrium and found differences in proliferation patterns between the uterine surface epithelium and endometrial basal glands. In addition, the authors reported a possible correlation between serum P4 levels and endometrial basal gland proliferation. Furthermore, a higher proliferative rate was found in the epithelia of a bitch’s uterus at late anestrus, when the level of estradiol (E2) was increasing while the level of P4 was still low and entering the next cycle [10, 11]. However, the previous studies by Galabova *et al*. [10] and Lindh *et al*. [11] have included only diestrus and anestrus stages. In addition to steroid hormones, steroid receptor proteins and endometrial proliferative activity have been reported. PR and Ki-67 levels were highest during proestrus and estrus, whereas they were low during other stages. A significant difference in Ki-67 immunostaining was observed only in the surface epithelium, and a positive correlation was also found between PR and Ki-67 positive cells in the surface epithelium [12]. However, there have been no published reports on uterine proliferation in late-term pregnant bitches. The apoptotic cell count or the apoptotic index (AI) can be considered to be a relationship between the percentage of tumor cells present and the opposite of proliferation [13]. Apoptosis can be detected by microscopy, conventional histopathology, or other special molecular techniques [13]. Terminal deoxynucleotidyl-transferase (TdT) mediated by 5’ deoxy-uridine-triphosphate is a frequently used method to detect apoptosis by labeling fragments of DNA. TUNEL is used to label the 3′-OH ends of DNA fragments. The 3′-OH ends resulting from DNA fragmentation were labeled with nucleotides modified by the TdT enzyme. This enzyme detects apoptosis more selectively than necrotic cells [13]. Proliferation and apoptosis play an important role in cyclic changes in normal canine endometrium during the estrus cycle. Morphological and functional changes in the canine endometrium were related to a progesterone-independent mechanism and apoptosis when luteal regression occurred in early anestrus [14]. Aglepristone, an antiprogestin acting as a PR blocker, has been indicated for the treatment of various progesterone-dependent physiologies, including induction of abortion, parturition, pathological conditions, including cystic endometrial hyperplasia, pyometra, acromegaly, insulin resistance, and mammary growth hormone-induced insulin-like growth factor-I secretion in bitches [15–17]. The recommended dose of aglepristone was 10 mg/kg subcutaneously (SC) with various repeated injection protocols depending on problems such as feline mammary gland hyperplasia, pyometra, mismating, and termination; however, the recommended dosage for planned cesarean section (C-section) was 15 mg/kg SC, a single injection at 24 h before planned C-section [15–17].

There have been many studies on aglepristone for parturition induction in bitches. Aglepristone interferes with hormone function at the PR and causes pregnancy loss. The same signaling cascade can also be applied to normal prepartum luteolysis [18]. Both natural and induced parturition, including antiprogestin treatment by withdrawal of the P4 cascade, result in disruption of the fetomaternal interface, alterations in vascular function, apoptosis, and promotion of a proinflammatory immune response [19]. Data on cell proliferation and apoptosis in the uterus at interplacental areas following aglepristone treatment in late pregnant bitch were limited.

The present study aimed to investigate the proliferation and apoptosis of uterine tissues at interplacental areas after a single aglepristone treatment (15 mg/mL, SC) in pregnant bitches scheduled 20–24 h before a planned C-section. The results of this study should provide more information on the mechanisms of aglepristone on uterine tissues that could be used as therapeutics for uterine disorders in bitches.

## Materials and Methods

### Ethical approval and informed consent

Signed consent was obtained from each owner for all animal samples in the study. All procedures were performed in accordance with the guidelines approved by the Kasetsart University Institutional Animal Care and Use Committee (approval number #UI-05952-2559) and by the Ethical Review Board of the Office of National Research Council of Thailand, Institute of Animals for Scientific Purpose Development.

### Study period and location

The study period was 24 months from May 2021 to April 2023. The study was conducted as a prospective study. Blood and uterine tissue, including all medical data, were collected from French bulldog bitches scheduled for planned C-section at the Kasetsart University Veterinary Teaching Hospital, Bangkok, Thailand and at the Obstetric Gynecology and Reproduction Clinic, Small Animal Teaching Hospital, Chulalongkorn University, Bangkok, Thailand. All laboratory procedures were performed at the Kasetsart University Veterinary Diagnostic Center, Bangkhen campus, Thailand.

### Animal and data collection from parturition bitches

The study was conducted as a clinical trial. Thirteen mature French bulldog bitches (*Canis lupus familiaris*) aged 1.5–7 years and weight 8–12 kg were enrolled in this study. Serum progesterone at 4–10 ng/mL and approximately 100% cornification cells from vaginal cytology examination were used for ovulation day prediction. Bitches were mated by artificial insemination 1–2 times between the 1^st^ and 4^th^ days after ovulation. Pregnancy was detected 28–35 days later on ultrasonography. A complete blood count, blood chemistry profiles, radiography, and ultrasonography were used to determine the health status of each bitch before random allocation to either the treatment or control groups. Agelpristone (Alizine^®^, Virbac, France) (15 mg/kg) was subcutaneously administrated into the treatment group on day 60 post-ovulation [15, 17]. Both the control and treated groups were manipulated according to the same protocol on day 60 of pregnancy, counted from ovulation day. Blood collection and ultrasonography were performed in the control and treated groups. In the control group, the vehicle of the drug (aglepristone) or normal saline was not treated because the owner preferred not to treat the bitches with any unnecessary drugs. Both treatment and control pregnant bitches were scheduled for cesarean delivery approximately 20–24 h later. Anesthetics, analgesics, and surgical protocols were followed according to the accepted standards.

#### Serum progesterone assay

For each dog, two blood samples (3 mL each) were collected from the cephalic vein 20–24 h before and at the C-section time. Serum samples were separated within 20 min of blood collection using centrifugation and stored at –80°C. The serum samples were sent to a certified laboratory, where P4 was measured using the chemiluminescence method (Immulite^®^, Siemens, USA). The assay precision, analytic sensitivity, and higher limit of detection were 10% (total coefficient of variation), 0.1 ng/mL, and 40 ng/mL, respectively.

#### Tissue collection

Small pieces of uterine horn samples were collected from the interplacental areas during C-section. Tissue samples (1 cm × 1 cm) were cut into small pieces and fixed in 4% (wt/vol) paraformaldehyde in phosphate-buffered saline for 48 h. Fixed tissue samples were embedded in a paraffin block, cut into sections (each 4 m thick), and placed on gelatin-coated slides for immunohistochemistry [20].

#### Proliferation: localization of Ki-67 antigen with immunohistochemistry

Sections of the uterine interplacental areas of each bitch were deparaffinized in xylene and rehydrated in graded ethanol. Heating with Tris ethylenediaminetetraacetic acid (pH = 9.0) at 95°C for 15 s was used as an antigen retrieval technique. The sections were arranged using the Novolink^®^ Polymer Detection Systems (Leica Biosystems, Newcastle Upon Tyne, UK). Briefly, they were immersed in 3% (v/v) hydrogen peroxide peroxidase to quench any endogenous peroxidase activity and then incubated with 0.4% casein protein blocking in phosphate-buffered saline (PBS) and 0.2% Bronidox L as a preservative. The sections with the monoclonal mouse anti-human Ki-67 antibody RTU (clone MIB-1) (Dako^®^, Glostrup, Denmark) were incubated at 37°C for 1 h [21] and washed in BOND^®^ Wash Solution (Novolink; Leica Biosystems, Newcastle Upon Tyne, UK). Subsequently, the sections were incubated at 37°C for 30 min with rabbit anti-mouse IgG (10 ug/mL) in 10% (v/v) animal serum in tris-buffered saline (TBS)/0.09% ProClin^®^ 950 (Novolink, Sigma Aldrich, USA). Finally, antibody binding was visualized using Novolink^®^ Polymer-anti-rabbit poly-horseradish peroxidase-IgG (25 ug/mL) containing 10% (v/v) animal serum in TRS/0.09% ProClin^®^ 950 and 1.74% W/V 3,3 diaminobenzidine chromogen (DAB). Mayer’s hematoxylin counterstain was used. We dehydrated the slides and mounted them with Neo-Mount^®^ (Merck-Chemicals, Darmstadt, Germany). The uterine sections without specific primary antibodies served as the negative control. Positive controls were the canine uterus in the diestrus stage, known to express Ki-67. Proliferation was evaluated using the Ki-67 labeling index (%) [22].

#### Apoptosis using TUNEL assay

Uterine sections of each bitch were deparaffinized in xylene, rehydrated in graded ethanol, and examined using the Apoptag^®^ peroxidase detection kit (Sigma Aldrich, Merck KGaA, Darmstadt, Germany) for TUNEL. Antigen retrieval was performed using proteinase K at 25°C for 15 min. The sample was then washed twice in PBS (5 min each time). Equilibration buffer was directly applied on the sections and incubated at 25°C for at least 5 min. TdT with working strength was applied in the sections and incubated in a humidified chamber at 37°C for 1 h. The sections were agitated for 15 s, incubated at 25°C for 10 min, and washed in PBS. Subsequently, the sections were incubated with anti-digoxigenin conjugate at 25°C for 30 min and washed with PBS. Finally, a positive brown color was visualized using the impact DAB kit stock (Leica Biosystems, UK). Mayer’s hematoxylin was used as a counterstain. We dehydrated the slides and mounted them with Neo-Mount^®^. Uterine sections in which the TdT enzyme was replaced by PBS were used as a negative control. A positive control was obtained from a canine uterus in the diestrus stage, which is known to present apoptotic cells. Apoptosis was evaluated on the basis of AI (%) [22].

#### Proliferation and AI

The uterine sections were imaged at 40×, 100×, and 400× magnifications using a DP73 camera (Olympus, Tokyo, Japan) attached to an optical BX53 microscope (Olympus), and the images were processed using the Olympus software. Proliferation and apoptosis were detected in four different tissue layers: uterine epithelium, stroma, glandular epithelium (uterine gland), and myometrium. Proliferation and apoptosis were evaluated as percentages of the Ki-67 labeling index (PI) and AI, respectively. Five interplacental areas were randomly selected, and 100 cells per region were evaluated per layer under a light microscope at 400× magnification. Positive Ki-67 staining and apoptosis were evaluated using %PI and %AI, respectively [22].

### Statistical analysis

Data are reported as mean ± standard deviation (SD) values. Comparisons of two mean values between groups were based on the Mann–Whitney U-test. A value of p = 0.05 was considered statistically significant. All statistical comparisons were performed using the RStudio software package (Version 1.0153–2009-2017, Integrated Development for R. RStudio, Inc., Boston, MA, USA), Rcmdr (R Commander, R package version 3.4.4.), and the R statistical analysis software package (R Core Team, 2017,www.r-poject.org).

## Results

### Animals

A total of 13 client-owned French bulldogs were enrolled in this study and divided into treatment (n = 8) and control (n = 5) groups. One bitch from the treatment group (T-No. 8) was excluded because of an emergency C-section 8 h after aglepristone administration.

### Serum progesterone levels

There were no significant differences in serum P4 levels between the treatment and control groups on the day of aglepristone administration (60-day post-ovulation), 20–24 h before C-section, and planned C-section day (61-day post-ovulation), as shown in Table-1.

**Table 1:** Mean ± SD and range of serum progesterone (P4) levels at 20–24 h before and at C-section time for treatment and control groups.

Group	Mean ± SD (range) of P4 level (ng/mL)	

	20–24 h before C-section	C-section time
Treatment (n = 7)	6.09 ± 2.72 (3.37–8.81)	4.32 ± 2.2 (2.12–6.52)
Control (n = 5)	5.45 ± 1.28 (4.17–6.73)	3.67 ± 1.89 (1.78–5.56)

SD=Standard deviation, C-section=Cesarean section

### Proliferation: localization of Ki-67 antigen with immunohistochemistry

**Figure-1:**
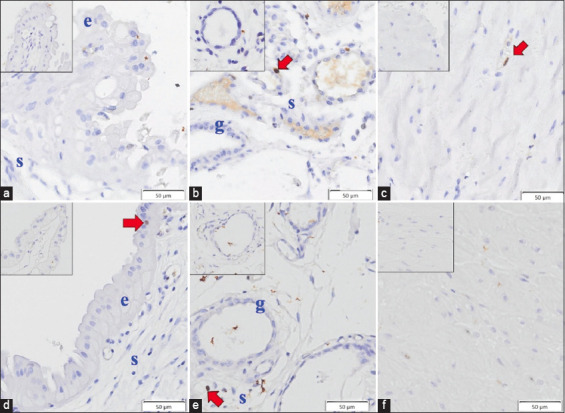
Immunohistochemical staining of Ki-67 in treatment (a–c) and control (d–f) groups of 4 different uterine tissue layers at inter-placental areas: e=Epithelium, s=Stroma, g=Glandular epithelium and myometrium (c and f). Positive cells are marked by red arrows, and negative controls are shown in insets (scale bar=50 μm).

**Figure-2:**
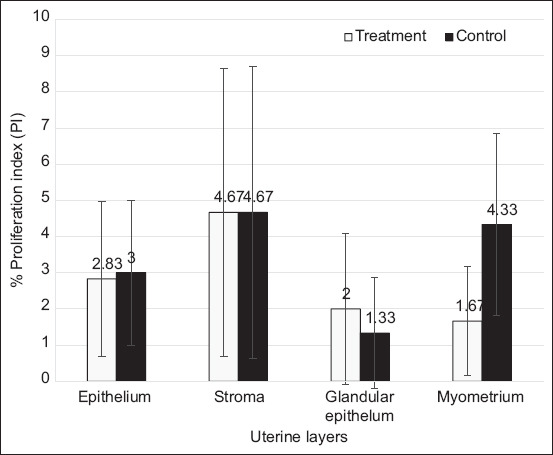
Mean ± SD of percentage of proliferation index of 4 layers in uterine tissue at inter-placenta areas (epithelium, stroma, glandular epithelium, and myometrium) from both treatment and control groups.

The four demonstrable uterine tissue layers at interplacental areas were the epithelium, stroma, glandular epithelium, and myometrium, with Ki-67 positivity in the treatment and control groups (Figure-1). No significant differences were observed in %PI between the groups. The %PI < 5% in the treatment (n = 5) and control (n = 3) groups (Figure-2).

### Apoptosis based on TUNEL assay

**Figure-3:**
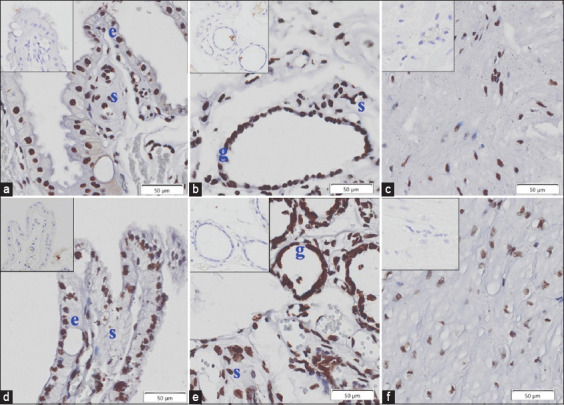
Apoptosis based on TUNEL assay in treatment (a–c) and control (d–f) groups of 4 different uterine tissue layers at inter-placental areas: e=Epithelium, s=Stroma, g=Glandular and myometrium (c and f). Positive cells are colored brown, and negative controls are shown in insets (scale bar = 50 μm).

**Figure-4:**
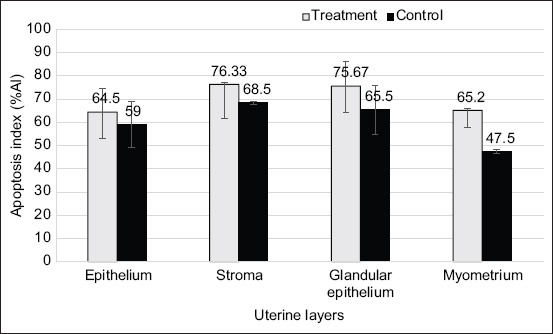
Mean ± SD of percentage of apoptotic index of uterine tissue in 4 layers at inter-placenta areas: Epithelium, stroma, glandular epithelium, and myometrium from both treatment and control groups.

Four uterine tissue layers, epithelium, stroma, glandular epithelium, and myometrium, were positive for apoptosis based on TUNEL assay in the treatment and control groups, as presented in Figure-3. There were no significant differences in apoptosis (%AI) between the treatment (n = 5) and control (n = 2) groups, with values higher than 45% (Figure-4).

## Discussion

Aglepristone 15 mg/kg SC administered 20–24 h before planned C-section in the bitches did not directly affect serum P4 levels. The results were similar to those of a previous study by Maenhoudt *et al*. [23] in which a single dose of aglepristone (10 mg/kg SC) was administered 5 days before ovariohysterectomy but did not affect non-pregnant diestrus bitches. Another study reported that aglepristone treatment did not affect either serum E2 or P4 levels [24]. In the present study, progesterone levels were lower at the time of C-section but were not below 2 ng/mL in both the treatment and control groups because all bitches underwent C-section before natural parturition time. No abnormal clinical signs were observed in the 12 bitches (excluded T-No.8) or in their puppies 7 days after C-section in either group. A single dose of aglepristone treatment did not affect the bitches or their puppies, similar to other studies [15, 24].

One treated bitch (T-No. 8) was excluded due to an emergency C-section after 8 h of aglepristone treatment because the P4 level naturally dropped below 2 ng/mL at the treatment time. According to the protocol, aglepristone was injected into the dog immediately after blood collection. Subsequently, P4 was analyzed and reported approximately 4 h after blood collection. Thus, aglepristone did not induce parturition of this bitch. Low P4 in this dog was due to natural parturition and not from the effect of aglepristone. The dog (T-No. 8) exhibited clinical signs of uterine contraction and mucoid vaginal discharge with no signs of delivered puppies. The owner sent the dog to an emergency C-section due to dystocia in the breed. The previous studies by Gogny and Fiéni [15] and Tavares Pereira *et al*. [25] have reported that aglepristone should be treated for a planned C-section in bitches 61 days after ovulation (59 days after the first mating date) before any stage of parturition. Another application reported to induce parturition involved combined injections of aglepristone (15 mg/kg, SC) and oxytocin (0.15 IU, SC) every 2 h until parturition [15].

P4 signaling in the canine placenta may regulate the vascular tonus, vascular integrity, invasive properties of the trophoblast, and decasualization. Vascular integrity will affect cell apoptosis [25]. One study on the effects of aglepristone treatment on placental expulsion in ewes found no significant difference (p > 0.05) in the number of apoptotic cells between the control group (88.5 ± 1.99) and the aglepristone treatment group (92.6 ± 1.48) with 5 mg/kg SC, twice, on days 140 and 141 post-mating. However, expulsion time of last lamb was prolonged in the treatment group [26]. Although the effects of P4 and PR included inhibition of transcriptional activity, negative regulation of proliferation and protein metabolism, and an increased immune response and apoptosis [27], the effect of PR blocker was unlikely to produce a sudden proliferation or apoptosis cells 20–24 h after administration. The results of the present study detected a very low PI% level (<5%) and a higher AI% level (>45%) at interplacental areas in both groups. This may be expected in natural uterine interplacental areas around the parturition period since all bitches had parturition within 62 days from the ovulation date. High apoptotic and low proliferating cells may be used for parturated preparation, endometrial regeneration, and uterine involution.

Sarli *et al*. [28] conducted a study on placental histology and neonatal outcomes. The placenta area contained a spot of necrosis; however, there have been no reports regarding the placental and interplacental areas of maternal uterine tissue. In this study, interplacental uterine tissues tended to undergo apoptosis. Apoptosis differs from necrosis in that apoptosis is a natural programed cell death with cell shrinkage, some characteristic morphological changes, and several enzyme-dependent biochemical processes without inflammation, whereas necrosis is an uncontrolled cell death with cell swelling and release of inflammatory cellular contents [29, 30]. Other techniques such as caspase 3, Bcl-2, and Bax proteins should be considered [14] to provide more information regarding uterine tissue apoptosis at the interplacental sites.

Tavares Pereira *et al*. [31] observed an expression pattern of several immune factors between natural and induced parturition based on aglepristone treatment in bitches to support the involvement of P4 signaling in the modulation of the uterine immune milieu. The study found that the infiltrate of macrophages involved in tissue remodeling was related to the downregulation of IL1β and -6, increased CD4+ lymphocytes, Treg, and Th, increased invasion of M1 macrophages and a higher incidence of NK cells in bitches treated with aglepristone for induced parturition. The difference in placenta maturation between induced and at term parturition may be due to the effects of aglepristone on P4 and PR signaling [31].

Further studies using antiprogestin to induce parturition in bitches at term should focus on proinflammatory interleukins and the uteroplacental immune system that may be related to apoptosis pathways.

## Conclusion

Aglepristone 15 mg/kg SC injected 20–24 h before a planned C-section did not affect P4, proliferation, and apoptosis of four layers of uterine tissue (epithelium, stroma, glandular epithelium, and myometrium) in the interplacental areas. The limitations of this study that low number of samples and other techniques such as caspase 3, Bcl-2, and Bax proteins should be considered to provide more information regarding uterine tissue apoptosis at the interplacental sites. Aglepristone is safe medicine for induced and planned C-section in bitches. Further study on uteroplacental immune system should be concerned.

## Authors’ Contributions

CL: Conducted the literature review, performed the study, interpreted the data verified the analytical methods, and drafted the manuscript. SP: Designed the study and drafted the manuscript. PT: Designed work on apoptosis laboratory part, analysis and interpretation of data and drafted the manuscript. PL: Designed work on the proliferation laboratory part, analysis and interpretation of data and drafted the manuscript. TS: Interpreted the data and verified the analytical methods. KS: Designed the study, the acquisition, and drafted the manuscript. All authors have read, reviewed, and approved the final manuscript.
